# Psychometric Properties of the Patient Self-Advocacy Scale: The Persian Version

**Published:** 2015-07

**Authors:** Shaghayegh Vahdat, Leila Hamzehgardeshi, Zeinab Hamzehgardeshi, Somayeh Hessam

**Affiliations:** 1 Department of Health Service Administration, Fars Science and Research Branch, Islamic Azad University, Marvdasht, Iran;; 2 Department of Health Service Administration, Marvdasht Branch, Islamic Azad University, Marvdasht, Iran;; 3 Public Health Center, Mazandaran University of Medical Sciences, Sari, Iran;; 4 Department of Reproductive Health and Midwifery, Nasibeh Nursing and Midwifery Faculty, Mazandaran University of Medical Sciences, Sari, Iran;; 5 Traditional and Complementary Medicine Research Centre, Mazandaran University of Medical Sciences, Sari, Iran;; 6 Department of Health Services Administration, Shiraz Branch, Islamic Azad University, Shiraz, Iran

**Keywords:** Psychometrics, Patients, Involvement

## Abstract

**Background:**

Advances in science and technology and the changes in lifestyle have changed the concept of health in terms of etiology and mortality. The aim of this study was to test the psychometric properties of the original Patient Self-Advocacy Scale for use with an Iranian population.

**Methods:**

In the current study, 50 chronic patients between the ages of 25 and 75 were selected as samples. This study was conducted in May 2013 at Bou Ali Sina Hospital in Sari. The translation process and cultural adaptation of the Patient Self-Advocacy Scale were conducted. The face validity and content validity of the instrument were formally verified by analyzing the feedback of patients and health professionals. In order to evaluate questionnaire’s reliability, the intraclass correlation coefficient (ICC) was calculated for each item and each domain; and the Cronbach’s alpha was calculated for the entire instruments and each domain.

**Results:**

Of the 50 patients participating in the study, 36% were male and 64% were female. The mean age of the patients was 42.5. To comply with the Iranian culture and the study target population, slight changes were applied to the process of translation and validation. In the present study, intraclass correlation coefficient for each item was 0.8-1, which demonstrates excellent reliability of the questionnaire. The Cronbach’s alpha value was 0.75 for overall scale.

**Conclusion:**

The Persian version of Patient Self-Advocacy Scale was valid and reliable. Hence, it can be used by public health researchers and health system policy makers for programming and offering patient-oriented health services based on patients’ comments, needs, and preferences.

## Introduction

In the recent decade, advances in science and technology and the changes in lifestyle have changed the concept of health in terms of etiology and mortality. Thus, infectious diseases have been controlled and replaced by chronic and metabolic diseases.^1^ The World Health Organization considers chronic diseases as the cause of 80% of deaths in low- to moderate-income countries.^2^ The increasing proliferation of chronic diseases, increased hospitalization due to the disease and the high cost of health care systems are the major challenges.^3^

The definition of contribution has been investigated in various studies. Brownlea defines participation as becoming engaged or to allow getting involved in decision-making process, service delivery, service evaluation, or even simply being in the position of an individual who is being consulted about a subject.^4^ The World Health Organization also defines participation as involvement in a life situation and considers learning and applying knowledge as the scope of activity and participation.^5^ Patients’ participation in health leads to greater satisfaction and trust, lower anxiety and stress, higher understanding of individual needs, positive and more efficient relationship of health professionals, and positive and permanent impacts on health.^6^

Several studies have shown the effect of patients’ involvement in health care with the improvement of chronic disease treatment outcomes. Participation of patients leads to improvement of diabetes control, physical functions in rheumatic diseases, drug dependence in depression, which all improve patient’s interest and commitment to undertake secondary prevention interventions. Thereby, they improve the health of patients suffering from myocardial infarction.^7-12^

Several tools have been constructed and validated to measure patients’ participation, including: Patient Self-Advocacy Scale (PSAS)^13^ and Control Preferences Scale (CPS).^14^ These tools have good reliability and validity. The PSAS is composed of 12 items answered in a 5-point Likert format, ranging from 1 (strongly agree) to 5 (strongly disagree). This scale consists of three domains: (1) increased illness education (four items); (2) increased assertiveness (four items), and (3) potential for mindful non-adherence (four items).^13^

Information about the disease and healthcare enables people to exchange mutual information with healthcare providers. Education essentially helps elevate individual’s audacity and willingness to ask questions, and this will lead to participation in health care decisions. Patients’ heightened knowledge and audacity, and their reasoning will result in non-compliance with health programs that are logically unacceptable to them.^15^ In the traditional compliance-gaining literature, patient non-adherence has been perceived by physicians as a form of deviance. However, Donovan and Blake asserted that non-adherence may not actually indicate deviance on the part of patients, but instead may represent reasoned decision-making based on rational choices regarding lifestyle choices and treatments drawn from patient beliefs, responsibilities, and preferences.^16^ In this sense, non-adherence can be viewed as “mindful” rather than “mindless”. Mindfulness is a state in which the individual consciously environmental cues. Applying this concept to adhere to treatment regimens, we can view non-adherence as a strategic or mindful action when it is based on contextual considerations (e.g., the patient’s own health beliefs or life circumstances). Through a desire for increased autonomy in their health care, activist patients are likely to have positive perceptions of and be willing to engage in instances of mindful non-adherence if they disagree with the efficacy of a physician’s treatment recommendations. In this case, instances of non-adherence behavior are not irresponsible or unreasonable but rather are carefully chosen actions based on the patients’ level of medical information about their disease and knowledge of their own personal health care needs and beliefs.^13^

A similar study was adapted based on the Patient Self-Advocacy Scale. The goal of the investigation was to adapt the Patient Self-Advocacy Scale in a sample of cancer survivors. Inter-factor correlations of the modified Patient Self-Advocacy Scale revealed results that follow closely with the original scale and results that indeed vary. The same three factors were maintained with nearly identical item loading. The split of one item into two different items resulted in both loading cleanly onto the expected factor (assertiveness). One difference was found with the reverse-coded item that read, “I don’t get what I need from my doctor because I am not assertive enough” which was loaded onto more than one factor and was omitted from the final scale. Reliability scores for the subscales were similar to the original scale with values (Pearson correlation and Cronbach’s alpha) ranging from 0.56 to 0.75 and overall scale Cronbach’s Alpha was 0.74.^17^ The aim of this study was to describe the process and principles used in the translation and cultural adaptation of PSAS in order to present the psychometric properties of the study for the first time in Iran.

## Patients and Methods

Permission to translate and use the PSAS was received from its designer, professor Brashers from the University of Illinois-Urbana/Champaign. The questionnaire was then translated and validated using forward-backward translation. At first, the questionnaire was separately translated from English into Persian by two Persian translators. Review and comparison of the two translations were then performed by two principle translators and researchers. At the third stage, the final translation was translated from Persian into English by two English translators who had no medical knowledge and had not seen the original version of the questionnaire. The English translated version was compared with the main tool, and the differences were discussed by the research team and the English translators. Eventually the final PSAS Persian version was prepared. In order to perform face validity and to determine the time required for completing the questionnaire, a pilot study at the Bou Ali Sina Hospital in Sari, May 2013 was conducted on 50 chronic patients aged 25-75. Written consent was obtained from the patients for participation in the study and they were assured their identities would remain confidential at all stages of investigation. The aim was to evaluate the face validity, reasonability, and attractiveness, reasonable sequence of items, clarity, brevity, and comprehensiveness of the tools in the eye of the target group (chronic patients).

Face validity was performed in two ways. The quality of face validity was evaluated to understand the relevance and relationship between items, the ambiguous and incomplete perceptions, and the patient’s difficulties in understanding the concepts. Furthermore, tool face validity was quantitatively assessed using impact score. Likert style was used for the scores. For each of the twelve items of the instrument, a 5-point Likert scale was considered in which “strongly important concerns” were scored as 5 and the answers of “not at all” were scored as 1. Item impact scores were calculated using a certain formula.^18^^,^^19^ Impact score equal or above 1.5 was identified as an important item.

Tool content validity was also measured qualitatively and quantitatively in order to ensure that the test content represents the factor it is measuring. In qualitative analysis of content validity, 10 health specialists were interviewed. They were also asked to carefully study the tools and offer their written corrective opinions. In the qualitative content validity, correct grammar, proper use of words, appropriate rating, and the time spent on completion of design tools, proportion of the selected dimensions and the like were considered by an expert panel of specialists. In order to evaluate quantitative content validity, content validity ratio (CVR) and content validity index (CVI) were used. CVR index indicates the importance of an item from the viewpoint of 10 specialists. CVI index was used to ensure 10 specialists that the tool items were designed in their best way to measure content.

Expert panel responded to each 12 items as ‘item is essential’, ‘item is useful but not essential’, or ‘item is not necessary’. Then CVR was calculated using a certain formula and according to Lawshe table, CVR>0.62 was recognized as criterion for essential items in the tool.^20^ Next, PSAS was given to the expert panel to comment on the clarity, relevancy and simplicity of each item in a 4-point Likert scale style. CVI was calculated using a certain formula and items were accepted as follows: CVI equal or above 0.79: means the item is adequate, 0.70<CVI<0.78 means the item is questionable and needs revision and correction, and CVI<0.70 means the item is unacceptable and should be eliminated.^21^

None of the items was considered unacceptable by using impact score, CVI and CVR. Based on the results of the pilot study, and experts’ comments, minor changes were made and PSAS-P was found appropriate for the Iranian society. At the next stage, test-retest and internal consistency were used to evaluate its reliability. To this end, 50 chronic patients aged 25-75 were selected. Of the 50 chronic patients: 10 patients with sexually transmitted diseases, 10 patients with gastrointestinal disease, 10 patients with renal disease and 20 patients with heart disease. They completed PSAS-P anonymously and after two weeks, the same group completed it again. Test-retest was used to evaluate the questionnaire’s reliability; the ICC was calculated for all domains and items in this study. Cronbach’s alpha was used to calculate internal consistency. Qualitative indexes for ICC were determined as follows: <0.4 (weak reliability), 0.4-0.6 (average reliability), 0.6-0.8 (good reliability), and 0.8-1 (excellent reliability).^22^ The statistical software used was SPSS 16.0.

## Results

Questionnaires were completed by participants within 10 minutes. Quantitative face validity results (impact score) showed that all 12 items (100%) impact scores over 1.5. Identifying these items indicated their importance of the target group’s (chronic patients) perspective. In measuring CVR, the score of 12 items (100%) was higher than that in Lawshe table (0.62 for 10 experts), which showed the importance and necessity of these items from the experts’ point of view.^18^ Furthermore, CVI score for the 12 items (100%) was greater than 0.79, and therefore, these items were found appropriate.

Based on the recommendations of the expert panel and the research team, as well as considering the Iranian culture, and the study target group, slight modifications were made in the instrument. In the domain of “increased illness education”, item 1 in the main tool asked of patients with HIV virus. Nevertheless, given the target group of the study, not only patients with HIV were investigated, but patients with a wide range of chronic diseases such as heart, renal, gastrointestinal, sexual diseases, and the like were also investigated, and eventually PSAS-P was constructed. To determine the reliability of the tool, 50 chronic patients with mean age of 42.5 years completed PSAS-P. Of the 50 chronic patients: 10 patients with sexually transmitted diseases, 10 patients with gastrointestinal disease, 10 patients with renal disease and 20 patients with heart disease. Of the patients, 36% (18) were men and 64% (32) women. In the present study, ICC for each item ranged 0.8 to 1, which indicates an excellent reliability for PSAS-P (table 1). ICC of each range was calculated separately (table 2). Internal consistency of overall scale was determined using Cronbach’s alpha. Cronbach’s alpha was calculated for each domain as follows: “increases illness education”=0.70, “increased assertiveness”=0.60, “potential for mindful non-adherence”=0.62 and “overall scale Cronbach’s Alpha”=0.75 (table 3).

**Table 1:** Reliability index in dimension of repeatability of PSAS-P questionnaire

**Items**	** ICC^a^**		
	**Mean **	**Minimum **	**Maximum **
Importance of information about disease and its treatment	0.95	0.86	0.98
Continuous search for information about disease	0.93	0.83	0.97
Acquiring more information in relation to healthy people about their health	0.86	0.64	0.94
Having full knowledge about similar people’s health problems	0.96	0.90	0.98
Lack of sufficient self-confidence to express care requirements	0.97	0.93	0.99
Having full confidence about care needs	0.93	0.83	0.97
Suggestion to health team about health needs	0.88	0.71	0.95
Questioning treatment methods, if not known or disagree	0.85	0.62	0.94
Having good reasons for non-compliance with care instructions	0.93	0.83	0.97
Having better understanding of health team in relation to meeting health needs	0.97	0.92	0.99
Non-acceptance of treatment in case of disagreement	0.96	0.90	0.98
Not following health care team’s instructions/suggestions	0.90	0.74	0.91

^a^Intraclass correlation coefficient

**Table 2:** Reliability index in dimension of repeatability of PSAS-P for each domain

**Domain number of study questions**		**ICC**		
		**Mean **	**Minimum **	**Maximum **
Increased illness education	4	0.86	0.74	0.93
Increased assertiveness	4	0.82	0.68	0.92
Potential for mindful non adherence	4	0.79	0.61	0.90

^a^Intraclass correlation coefficient

**Table 3:** Reliability index in dimension of internal consistency of PSAS-P questionnaire for the whole tool and domains

**Domain**	**Number of questions **	** CA^a^**
Increased illness education	4	0.70
Increased assertiveness	4	0.60
Potential for mindful non adherence	4	0.62
Total scale	12	0.75

^a^Cronbach’s Alpha

## Discussion

In this study, psychometrics of PSAS-P was examined. There were no serious difficulties in the process of translation and cultural adaptation of PSAS. Thus, other than minor changes, fundamental modification of the original version was not required. For instance, in the original version, target group consisted of HIV patients only, but in the present study, a wide range of patients with chronic diseases was included. In other words, there was a close correlation between the Persian and the English versions.

The validity and reliability of the tool were investigated. In order to evaluate validity, face and content validity was used. Face validity is mainly associated with understanding the text by the target group. A measuring tool must be clearly understood by the target group in order to ensure their cooperation, and motivate them for answering questions.^23^ Chronic patients expressed their opinions about the suitability and coherence of questions and understanding of concepts.

In content validity, the contents of the questionnaire were scrutinized. A questionnaire has content validity when questions are tested.^24^ First, the contents were examined by a panel of experts, and after careful study, they recommended their comments in writing. An advantage of this study was careful qualitative and quantitative assessment of face and content validity of the questionnaire. Each one of the quantitative and qualitative methods offers different but complementary perspectives of the study; this is the benefit of using combined methods in evaluating the questionnaire validity.^21^

In the present study, to assess face validity of the tool, quantitative method of the impact score index was used. The impact score of every item was calculated using the relevant formula, and was found to be higher than 1.5. This meant that, items had been considered suitable for further analysis. In assessing content validity, quantitative CVR and CVI indices were used. CVR index for each item was higher than that in the Lawshe table (0.62 for 10 for experts), which showed the importance and necessity of items in view of the experts. For every item, CVI score was greater than 0.79, and thus, items were considered suitable. 

The results indicated satisfactory reliability of PSAS-P. To assess PSAS-P reliability, test-retest and internal consistency methods were used. Cronbach’s alpha for the overall scale and for each domain was calculated and the internal consistency of overall scale was acceptable (Cronbach’s Alpha=0.75). The overall scale Cronbach’s alpha for the original version of PSAS was 0.78 and for “increased illness education”, “increased assertiveness”, and “potential for mindful non-adherence” were 0.64, 0.70, and 0.79, respectively.^13^ Cronbach’s alpha of 0.7 and higher were acceptable.^25^^,^^26^ In this study, Cronbach’s alpha for “increased assertiveness” and “potential for mindful non-adherence” were 0.60 and 0.62, respectively. According to a study by Carrico, Cronbach’s alpha of 0.6 and higher were acceptable.^27^ In assessing the reliability of the questionnaire in the dimension of repeatability (test-retest reliability), questionnaires were completed by participants within 2 weeks interval. ICC was calculated for all domains and items. ICC for “increased illness education, and “increased assertiveness” was in the range of 0.8-1, and for “potential for mindful non-adherence” ranged between 0.6-0.8. In addition, ICC for each of the items ranged 0.8-1.

According to researchers searching the databases available, a similar study adapted the Patient Self-Advocacy Scale .The results obtained in this study are comparable with the other study. Carol reported that with consideration for the target group, modifications had been implemented in the tool, and the reliability of the tool in the dimension of internal consistency was examined through the calculation of Cronbach’s alpha for the overall scale and each of the domains. The Cronbach’s alpha for overall scale=0.74, and for “increased assertiveness” and “potential for mindful non-adherence” equaled 0.67 and 0.75, respectively. To examine the reliability of “increased illness education” Pearson correlation was used, and found to be 0.56.^17^

## Conclusion

PSAS-P was produced through translation and cultural adaptation of PSAS original version. The results proved substantial changes to the original version were not necessary, and only minor changes were implemented. Internal consistency of PSAS-P of overall scale was acceptable, and the reliability of the tool in the dimension of repeatability (test-retest reliability) using ICC was 0.92. PSAS-P showed satisfactory quantitative and qualitative validity, and questions were clear and understandable by the patients. This encouraged them to take part in a pilot study and to answer questions. Therefore, PSAS-P can be used by public health researchers and policy makers in the health system for planning and the provision of patient-oriented health services based on patients’ views, needs, and preferences. Study limitations were that the studies were not done to test the psychometric properties of the original Patient Self-Advocacy Scale. Future studies on larger populations are required. 
